# Genetic and Molecular Basis for Heat Tolerance in Rice: Strategies for Resilience Under Climate Change

**DOI:** 10.3390/plants14223492

**Published:** 2025-11-16

**Authors:** Wei Zhang, Liang Zhou, Dewen Zhang

**Affiliations:** Anhui Province Key Laboratory of Rice Genetics and Breeding, Rice Research Institute, Anhui Academy of Agricultural Sciences, Hefei 230031, China; zhangw@aaas.org.cn (W.Z.); zhouliang@aaas.org.cn (L.Z.)

**Keywords:** rice, heat tolerance gene, molecular mechanisms, molecular breeding

## Abstract

Heat stress has emerged as a significant abiotic constraint affecting rice yield and grain quality. In recent years, substantial advancements have been achieved in elucidating molecular regulatory mechanisms and breeding applications pertinent to rice heat tolerance. This review offers a comprehensive examination of the fundamental regulatory pathways involved in rice responses to heat stress, encompassing membrane lipid homeostasis, heat signal transduction, transcriptional regulation, RNA stability and translation, epigenetic modifications, hormone signaling, antioxidant defense, and the protection of reproductive organs. Particular emphasis is placed on the functional mechanisms and breeding potential of pivotal thermotolerance-associated genes and quantitative trait loci (QTLs), such as *TT1*, *TT3*, and *QT12*. Additionally, we summarize recent applications of cutting-edge technologies in the enhancement of heat-tolerant rice varieties, including multi-omics integration, *CRISPR/Cas9* genome editing, marker-assisted selection (MAS), and rational design breeding. Finally, we address current challenges, including integrating regulatory mechanisms, developing realistic heat simulation systems, validating the functionality of candidate genes, and managing trait trade-offs. This review provides a theoretical foundation for developing heat-tolerant rice cultivars and offers valuable insights to accelerate the breeding of climate-resilient rice varieties for sustainable production.

## 1. Introduction

In the context of global climate warming, the Earth’s average surface temperature has increased by approximately 1.1 °C relative to pre-industrial levels [1]. Notably, from 2023 to 2025, an unprecedented temperature anomaly was recorded, with global average temperatures rising to 1.52 °C above pre-industrial levels, establishing a new historical record, as reported by the World Meteorological Organization and the European Centre for Medium-Range Weather Forecasts. For instance, in early July 2025, extreme heat events with daily maximum temperatures surpassing 39 °C were persistently observed in the middle and lower reaches of the Yangtze River in China. Climate model projections indicate that if greenhouse gas emissions continue on the current trajectory (RCP8.5 scenario), global surface temperatures could rise further by 1.5–4.5 °C by the end of this century [1], significantly increasing the frequency and intensity of extreme heat events. This trend poses a substantial threat to rice (*Oryza sativa*) production, particularly during critical growth stages such as flowering and grain filling. Heat stress during these periods impairs pollen viability by approximately 29% and suppresses the activity of key enzymes involved in starch biosynthesis (e.g., ADP-glucose pyrophosphorylase, AGPase)—in some processes reducing activity by up to 67%—ultimately resulting in reduced seed set and yield losses [2,3]. Previous studies have demonstrated that heat priming treatments can induce molecular and biochemical adjustments in rice, such as enhanced antioxidant activity and protein stability, which subsequently improve plant adaptation to heat stress [2,3]. Recent simulation studies suggest that if the current high-emission scenario (RCP8.5) continues, chronic heat stress could lead to global annual rice yield losses exceeding 11.4 million tons by 2050, with over 77% of these losses projected to occur in South and Southeast Asia [4]. This would severely impact regional food security and may indirectly affect global rice prices and accessibility.

Heat stress exerts significant stage-specific impacts on rice development, with reproductive phases such as panicle initiation, flowering, and grain filling being particularly susceptible [5]. Research indicates that continuous exposure to temperatures exceeding 35 °C for six hours during the heading stage can diminish pollen viability by over 29%, thereby severely impairing the pollination and fertilization processes [6]. During the grain-filling phase, extended heat stress markedly increases grain chalkiness and disrupts the equilibrium between protein and starch synthesis, ultimately resulting in reduced grain quality and yield [7]. Further mechanistic investigations have elucidated the molecular regulatory pathways through which heat stress influences grain development. For instance, under elevated temperatures, the heat-induced expression of the *QT12* gene inhibits the phosphorylation of starch synthase *SSIIIa* (resulting in decreased enzymatic activity) while simultaneously upregulating the expression of the storage protein transcription factor *RISBZ1*. This alteration leads to an approximate 28% reduction in amylose content and a 15% increase in the proportion of glutelin within the endosperm, thereby disrupting the homeostasis of storage substances, increasing chalkiness, and degrading eating quality [8]. Additionally, the *TT3* module contributes to heat tolerance by stabilizing chloroplasts. Specifically, *TT3.2* encodes a chloroplast-localized chaperone that directly protects photosystem II (PSII) complexes, enhancing CP43 stability by 40%. Simultaneously, *TT3.1* encodes an E3 ubiquitin ligase that facilitates the removal of misfolded proteins accumulated in the cytoplasm, thus maintaining chloroplast structure and function. These mechanisms enhance photosynthetic efficiency by up to 30% under heat stress [9]. Consequently, heat stress not only compromises physiological processes during the reproductive stage but also disrupts carbon–nitrogen metabolism, nutrient transport, and the accumulation of storage compounds during grain filling. These disruptions impact yield formation and quality stability across multiple biological levels [10].

Rice holds high importance for global food security and contributes substantially to the national income of major rice-producing countries [11,12]. Given the dual challenges posed by global climate change and threats to food security, achieving a comprehensive understanding of the genetic foundations and molecular regulatory mechanisms underlying rice heat tolerance has emerged as a leading focus in contemporary crop biology and genetic breeding research [13]. Recent advancements in multi-omics technologies, including genome-wide association studies (GWASs), transcriptomics, metabolomics, and epigenomics, have facilitated the identification and characterization of several key genes and quantitative trait loci (QTLs) associated with rice heat tolerance, such as *TT1* [14], *TT2* [15], *TT3* [9], and *QT12* [8]. Concurrently, the preliminary application of molecular marker-assisted selection (MAS) and *CRISPR/Cas9* gene-editing technologies in breeding programs has provided substantial support for the development of heat-tolerant rice varieties [16,17].

Currently, research on rice heat tolerance faces several challenges: (1) the efficiency of functional validation for candidate genes is suboptimal, with laboratory results frequently diverging from field performance; (2) there are substantial trade-offs between heat tolerance and agronomic traits, such as yield and quality, complicating simultaneous breeding for multiple traits; and (3) the application of rice heat tolerance genes is limited, hindering the practical translation of research outcomes.

This paper provides a systematic review of the molecular regulatory networks involved in rice heat tolerance, integrating recent research advancements to address existing challenges. This review emphasizes heat sensing and membrane homeostasis regulation, antioxidant systems, membrane lipid regulation, key quantitative trait loci (QTLs), and functional gene analysis. We also examine the crosstalk mechanisms between heat tolerance and grain development. Furthermore, we discuss the latest progress in the application of emerging gene-editing technologies, such as *CRISPR/Cas9*, and the development of breeding strategies designed to enhance rice heat tolerance. We conclude by identifying future research directions and highlighting the core issues that need to be addressed. In this review, we summarize the molecular mechanisms underlying rice heat tolerance across multiple developmental stages, including the vegetative, reproductive, and grain-filling phases. While heat sensitivity exhibits clear stage specificity, the studies discussed here collectively encompass diverse growth stages to provide a comprehensive understanding of heat response and adaptation mechanisms in rice.

## 2. Advances in Understanding the Molecular Mechanisms Behind Rice Heat Tolerance

### 2.1. Heat Sensing and Signal Transduction Mechanisms

**Figure 1:**
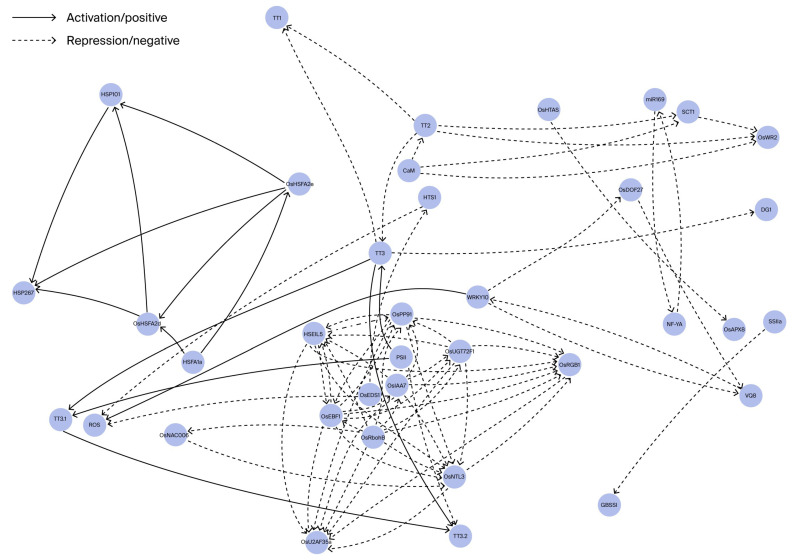
Membrane sensing and ROS regulation. *HTS1* is involved in maintaining chloroplast membrane stability under heat stress by modulating lipid remodeling and reducing reactive oxygen species (ROS) accumulation. The regulatory network shown in Figure 1 was manually assembled based on published studies summarizing experimentally validated genes involved in rice heat tolerance rather than generated from a database.

Rice detects high-temperature stress through a complex and coordinated regulatory system. Research has identified the *HTS1* gene, which encodes a β-ketoacyl carrier protein reductase located on the chloroplast membrane, as a crucial component in heat sensing (see Table 1 for a summary). Loss-of-function mutants of *hts1* demonstrate significant disruption to fatty acid synthesis, excessive accumulation of reactive oxygen species (ROS), and abnormal increases in cytosolic Ca^2+^ influx rates, ultimately resulting in compromised plasma membrane integrity and the initiation of programmed cell death (PCD) (Table 1) [18]. At the molecular regulation level, the RING-type E3 ubiquitin ligase *OsHTAS* plays a pivotal role in enhancing transport regulation efficiency by mediating H_2_O_2_ homeostasis and controlling stomatal movement (see Table 1 for a summary). Plants overexpressing *OsHTAS* exhibit improved water retention capacity and enhanced antioxidant defense mechanisms under heat stress conditions [19]. These findings not only elucidate the molecular connection between membrane lipid homeostasis and heat signal perception but also support the following multi-level heat-sensing network model: “phytochrome conformational changes → regulation of chloroplast membrane lipid unsaturation → ROS signal transduction → stomatal response”. To visualize the interconnected molecular pathways identified in this study and previous works, we constructed a comprehensive regulatory network (Figure 1).

Antioxidant Defense and Stomatal Regulation: The *OsHTAS–OsAPX8* module promotes H_2_O_2_-mediated stomatal closure while enhancing antioxidant capacity; *OsEDS1* maintains H_2_O_2_ homeostasis; *OsRbohB* functions as an NADPH oxidase-generating ROS; *PSL50* suppresses ROS overaccumulation; *OsMDHAR4* negatively regulates H_2_O_2_-induced stomatal closure; *OsProDH* promotes ROS accumulation; and *OsMYB55* enhances amino acid metabolism, contributing to thermotolerance. HSF Core Module and Molecular Chaperones: HSFA1a acts upstream to activate *OsHSFA2d/e*, which in turn induces *HSP101* and *HSP26.7* expression, constituting the classical heat shock response. The HSF–miR169–NF-YA feedback loop fine-tunes HSF activity. The *OsNTL3* → *OsbZIP74* endoplasmic reticulum (ER) stress axis forms a positive feedback module under heat stress. Regulatory Cofactors: *OsDOF27* positively regulates heat stress responses; the *WRKY10–VQ8* module functions antagonistically, with *WRKY10* acting as a negative regulator and *VQ8* inhibiting its DNA-binding activity. Protein Homeostasis: *TT1*, encoding the α2 subunit of the 26S proteasome, safeguards proteasome assembly and facilitates the degradation of misfolded proteins under heat stress. Chloroplast Protection and Cuticular Wax Biosynthesis: *TT3.1* promotes the degradation of *TT3.2* to protect PSII from heat-induced photoinhibition; the *TT2–CaM–SCT1–OsWR2* cascade enhances cuticular wax deposition, reducing heat-induced water loss.

### 2.2. HSF-Mediated Multi-Level Regulatory Mechanisms of Heat Tolerance in Rice

Heat shock transcription factors (HSFs) form the central component of the transcriptional regulatory network essential to rice thermotolerance. Within this group, *OsHSFA2e* and *OsHSFA2d* function as critical regulators that specifically bind to heat shock elements (*HSEs*) and jointly activate the expression of molecular chaperone genes, such as *HSP26.7* and *HSP101*, thereby facilitating the classical heat shock response (*HSR*) pathway [38]. Recent research has elucidated several key HSF-centered regulatory modules, including the *HSF–miR169–NF-YA* negative feedback loop, wherein HSFs transcriptionally activate *miR169*, which in turn represses NF-YA gene expression, creating a feedback mechanism that finely tunes the transcription of downstream HSF target genes [39]. Additionally, the *OsNTL3–OsbZIP74* ER stress axis has been characterized; during heat stress, the membrane-bound NAC transcription factor *OsNTL3* undergoes proteolytic cleavage and translocates to the nucleus, where it induces *OsbZIP74* expression, establishing a positive feedback loop that enhances endoplasmic reticulum (ER) stress tolerance [22]. Furthermore, hormonal crosstalk via abscisic acid (ABA) biosynthesis has been identified, wherein *HSFA2s* not only regulate *HSP* gene expression directly but also promote the transcription of genes involved in ABA biosynthesis. *HSFA2* proteins not only directly regulate the expression of *HSP* genes but also enhance the transcription of genes involved in abscisic acid (ABA) biosynthesis. This dual function modulates the stomatal aperture and integrates heat response into hormonal signaling pathways [40,41].

### 2.3. Regulation of Heat Response by ALBA Proteins via mRNA Stabilization Mechanism

ALBA proteins, including *ALBA4*, *ALBA5*, and *ALBA6*, play a crucial role in enhancing plant tolerance to elevated temperature stress by stabilizing the mRNAs of heat shock transcription factors (*HSFs*). The underlying mechanism is as follows: heat stress triggers liquid–liquid phase separation (*LLPS*) in ALBA proteins, resulting in the formation of cytoplasmic stress granules (SGs) and processing bodies (PBs). *ALBA4*, *ALBA5*, and *ALBA6* directly bind to *HSF* mRNAs, sequestering them within SGs and PBs, thereby protecting them from degradation by the exonuclease *XRN4* and ensuring that HSF mRNAs remain stable. Consequently, these stabilized mRNAs are efficiently translated, leading to the production of heat shock proteins (HSPs) that confer cellular thermoprotection and facilitate growth recovery. Triple mutants lacking *ALBA4*, *ALBA5*, and *ALBA6* exhibit heightened sensitivity to high temperatures, whereas the knockout of *XRN4* partially restores heat tolerance, underscoring the significance of this pathway. This discovery unveils a novel function of ALBA proteins in plant responses to abiotic stress and underscores the importance of post-transcriptional mRNA stabilization through phase separation as a vital component of the heat shock response.

### 2.4. Epigenetic Regulation, Chromatin Remodeling, and Transcriptional Coregulation Comprise the Heat Response Expression Framework

The capacity of rice to rapidly and consistently respond to high-temperature stress is fundamentally supported by precise epigenetic regulation and chromatin remodeling, which orchestrate the transcriptional activation of heat responsive genes. Exposure to elevated temperatures results in DNA hypomethylation within the promoter regions of crucial thermotolerance genes, including *OsHSPs* and *OsMYB55*. This is accompanied by an enrichment of activating histone markers (*H3K4me3*, *H3K27ac*) and a concurrent reduction in the repressive marker *H3K27me3*. These chromatin modifications lead to a more open chromatin configuration, thereby enhancing the accessibility of transcription factors and facilitating the expression of heat-inducible genes [42].

A critical aspect of this dynamic regulation is the heat-induced remodeling of the histone variant *H2A.Z*. Elevated temperatures promote the selective splicing of *H2A.Z* from the promoter regions of heat-responsive genes, thereby exposing an essential cis-element and significantly enhancing the binding efficiency of master regulators such as *HSFA1a*. This “nucleosome–transcription factor” interaction accelerates the initiation and amplification of transcriptional activation during the early stages of heat sensing [43,44].

Recent research has advanced this framework by elucidating a functional interaction between histone acetylation and RNA Polymerase II (Pol II) phosphorylation. In *Arabidopsis*, the bromodomain proteins GTE2 and GTE7 are involved in chromatin remodeling and transcriptional regulation under heat stress, providing insights that may be applicable to understanding similar mechanisms in rice. During heat stress, this recruitment enhances the phosphorylation of the Pol II C-terminal domain (CTD) at Ser2 and Ser5, thereby maintaining the transcriptional elongation of heat-responsive genes [45]. The GTE2/GTE7–CDKL9 complex is extensively enriched by the promoters of stress-responsive genes during heat exposure, thereby protecting the stability of the transcriptional machinery. Mutants with loss-of-function in *gte2*/*gte7* or *cdkl9* exhibit heightened sensitivity to heat, highlighting the physiological importance of this regulatory pathway. Within the epigenetic–transcriptional framework, auxiliary transcriptional modulators play a critical role in refining the heat response. *OsDOF27*, an intrinsically disordered protein (IDP), enhances thermotolerance, as evidenced by overexpression lines that demonstrate approximately double the survival rate of wild-type (WT) plants after 24 h at 45 °C, without incurring any detectable growth penalties under optimal conditions. The *WRKY10–VQ8* module further exemplifies this regulatory complexity: WRKY10 functions as a transcriptional repressor by promoting the accumulation of reactive oxygen species (ROS) and accelerating programmed cell death under elevated temperatures. In contrast, *VQ8* interacts physically with *WRKY10*, inhibiting its DNA-binding activity and thereby suppressing the expression of downstream pro-senescence genes (*NAC4*, *OxO4*, *SGR*), ultimately enhancing survival. These epigenetic and transcriptional coregulation mechanisms establish a multi-layered expression framework in rice. This framework integrates chromatin remodeling, histone modifications, nucleosome dynamics, and transcription factor networks, supported by modulators such as *OsDOF27* and the *WRKY10–VQ8* complex, to collaboratively ensure a robust and adaptive response to heat stress.

### 2.5. Regulation of Heat Tolerance via Membrane Homeostasis and Antioxidant Networks

Membrane integrity and redox balance constitute two interdependent pillars of the rice heat tolerance system. Elevated temperatures pose a challenge to membrane stability by altering lipid composition and increasing the production of reactive oxygen species (ROS), which, if left unchecked, can result in irreversible damage not only to cells but also to DNA. Rice counteracts these effects through the coordinated regulation of membrane lipid homeostasis and antioxidant defense pathways.

A critical component of membrane lipid regulation is *HTS1*, a gene encoding a β-ketoacyl carrier protein reductase localized to the chloroplast thylakoid membrane. *HTS1* is instrumental in fatty acid biosynthesis, thereby influencing the degree of membrane lipid unsaturation. Under conditions of heat stress, *hts1* mutants exhibit significantly reduced unsaturation, excessive ROS accumulation, and increased Ca^2+^ influx, leading to membrane rupture, chloroplast degradation, and intensified programmed cell death (PCD). These mutants also show suppressed activation of downstream heat stress signaling pathways [18]. Therefore, maintaining an optimal unsaturated-to-saturated fatty acid ratio in chloroplast membranes is essential to photosystem stability and thermal resilience. In addition to membrane stabilization, various regulatory mechanisms enhance antioxidant defenses. *OsEDS1* serves as a positive regulator of thermotolerance by maintaining hydrogen peroxide (H_2_O_2_) homeostasis. Mutants lacking *OsEDS1* exhibit reduced survival rates (33.6% compared to 73.4% in wild-type plants), increased ion leakage, and diminished chlorophyll content under heat stress conditions. However, the complementation of the gene restores heat tolerance [26]. *OsHTAS*, a RING-type E3 ubiquitin ligase, facilitates stomatal closure by modulating H_2_O_2_ accumulation, thereby enhancing transpirational regulation. It physically interacts with the antioxidant enzyme *OsAPX8*, forming a synergistic reactive oxygen species (ROS)-scavenging module. Lines overexpressing *OsHTAS* demonstrate increased survival and enhanced antioxidant capacity under heat stress [19]. *PSL50* acts as a positive regulator of thermotolerance by reducing ROS accumulation and preventing cell death; mutants lacking *PSL50* are sensitive to heat but can partially recover tolerance under high-light conditions [27]. Conversely, *OsProDH* negatively regulates thermotolerance; gene knockout results in increased survival, whereas overexpression leads to heightened heat sensitivity [28]. *OsMDHAR4* functions as a negative regulator of hydrogen peroxide (H_2_O_2_)-induced stomatal closure, with loss-of-function mutants demonstrating improved survival under heat stress conditions [29]. *OsMYB55* contributes to enhanced thermotolerance by facilitating amino acid biosynthesis, thereby sustaining cellular metabolism during heat stress; transgenic lines with *OsMYB55* overexpressed exhibit reduced yield loss under elevated temperatures [25].

Additional genetic components, such as *OsRGB1* [46], the *QT12* QTL module, *OsNTL3*, *OsEBF1–OsEIL5–OsPP91* [47], *OsUGT72F1* [48], *OsU2AF35a*, *OsIAA7*, *SCE1*, and *OsRbohB*, are involved in various processes, including signal transduction, protein modification, antioxidant defense, sugar metabolism, and RNA splicing, thereby enhancing the complex network of heat tolerance. It elucidates a highly integrated system where membrane lipid unsaturation preserves structural integrity, while coordinated antioxidant mechanisms mitigate reactive oxygen species (ROS) and maintain cellular homeostasis. This interaction between membrane biology and redox regulation constitutes a central axis in the rice heat stress response.

## 3. Overview of Key Genes Involved in Rice Heat Tolerance

In recent years, the application of advanced methodologies, including genome-wide association studies (GWASs), transcriptome analysis, bulked segregant analysis sequencing (BSA-seq), and map-based cloning, has enabled researchers to systematically identify and characterize a series of key genes and quantitative trait loci (QTLs) integral to the regulation of heat tolerance in rice. These genes are implicated in various regulatory mechanisms, encompassing heat signal perception, membrane system homeostasis, transcriptional regulation, chloroplast protection, and grain development. Notably, genes such as *QT12* [8], *TT1* [14], and *TT3* [9] are active at different developmental stages in rice, such as booting, flowering, and grain-filling stages, enhancing heat tolerance by modulating transcriptional responses, protein homeostasis, and hormone transport.

### 3.1. TT1- and TT2-Mediated Regulation of Heat Tolerance in Rice

Both *TT1* and *TT2* contribute to rice thermotolerance, but they differ in mechanistic emphasis and degree of characterization. *TT1*, mapped to chromosome 3 and associated with the candidate gene *LOC_Os03g0387100* (encoding the α2 subunit of the 26S proteasome, also known as *OsPAB1*), has been well characterized. *TT1* enhances intracellular protein homeostasis under elevated temperatures by accelerating the degradation and recycling of heat-damaged proteins, thereby mitigating protein aggregation and protecting reproductive development. Genetic analyses indicate beneficial *TT1* alleles in African rice (*Oryza glaberrima*), suggesting an evolutionary role in heat adaptation [14]

Both *TT1* and *TT2* contribute to rice thermotolerance, but they differ in mechanistic emphasis and degree of characterization. In the domain of crop thermotolerance research, Thermo-tolerance 1 (*TT1*) represents the inaugural quantitative trait locus (QTL) successfully identified and cloned in relation to heat tolerance. Situated on chromosome 3, *TT1* is associated with the candidate gene Os03g0387100, which encodes the α2 subunit of the 26S proteasome, also referred to as *OsPAB1*. The 26S proteasome is a vital protein degradation complex responsible for maintaining cellular proteostasis by eliminating misfolded proteins and modulating stress-responsive pathways. The α2 subunit plays a pivotal role in the stability of proteasome assembly and its regulatory function during heat stress.

Functional characterization has revealed that *TT1* enhances intracellular protein homeostasis under elevated temperatures by expediting the degradation and recycling of heat-damaged proteins, thereby mitigating protein aggregation. Genetic analyses suggest that advantageous alleles of *TT1* are present in African rice (*Oryza glaberrima*), which demonstrates significantly greater heat adaptability compared to Asian cultivated rice (*O. sativa*). This interspecific variation implies that *TT1* has been instrumental in the evolutionary adaptation of rice to high-temperature environments.

The cloning and functional characterization of *TT1* have yielded critical mechanistic insights into plant heat response mechanisms and have also provided a valuable genetic resource for the molecular breeding of heat-tolerant rice varieties [14]. In addition to *TT1*, other key genes such as *RBG1* have also been shown to influence heat resilience and yield potential which will be further discussed in the proceeding section of this review. *TT2*, by contrast, encodes a G protein γ subunit that modulates cytosolic Ca^2+^ signaling and regulates cuticular wax deposition under heat stress. Mechanistically, *TT2* mediates the interaction between calmodulin (CaM) and the transcription factor *SCT1*, which represses *OsWR2* transcription; loss of *TT2* attenuates heat-induced Ca^2+^ increases and derepresses *OsWR2*, maintaining wax accumulation and thereby contributing to thermotolerance[15].

Notably, TT2 remains the least characterized among the TT QTLs. This relative paucity of studies may reflect several factors: (i) its action via cuticular wax metabolism is an indirect route to thermotolerance that often yields subtle or environment-sensitive phenotypes; (ii) characterized alleles or mutant resources are limited; and (iii) experimental analysis of wax composition and cuticle ultrastructure requires specialized biochemical and microscopic approaches. We therefore consolidated TT2 into this section and suggest targeted future directions (e.g., allelic surveys, tissue-specific functional assays, and integrated biochemical–phenotypic analyses) to better define TT2’s contribution to rice heat resilience.

### 3.2. The TT3 Genetic Module Enhances Heat Tolerance in Rice by Regulating PSII Stability

Under conditions of heat stress, the plasma membrane-localized E3 ubiquitin ligase *TT3.1* relocates to the endosome, where it co-localizes with the chloroplast precursor protein *TT3.2*. *TT3.1* ubiquitinates *TT3.2*, directing it towards degradation via multivesicular bodies and the vacuolar degradation pathway, which consequently reduces the accumulation of mature *TT3.2* protein in the thylakoid membrane of chloroplasts. An excessive accumulation of *TT3.2* compromises the stability of the photosystem II (PSII) complex and damages the thylakoid structure, resulting in a heat-sensitive phenotype. Conversely, the high-activity allele *TT3.1* efficiently facilitates *TT3.2* degradation, thereby enhancing chloroplast resistance to heat stress. During the reproductive stage, this regulatory module significantly improves rice heat tolerance; the overexpression of *TT3.1* or knockout of *TT3.2* results in an increase in per-plant yield of 26–38% and substantially enhances the seed setting rate and 1000-grain weight [9].

### 3.3. QT12, a Regulator of Heat Response Affecting Grain Quality and Yield

In an F_2_ population derived from the cross between *Chenghui448*, which exhibits minimal chalkiness increase under high temperature, and *OM1723*, which shows a substantial increase, the *QT12* locus was identified. The natural variation in *QT12* between these two parental lines is attributed to a G-to-A single-nucleotide polymorphism (SNP) located approximately 3 kb upstream in the promoter region. Seven promoter variants were identified, including a G-to-A SNP within a CCAAT-box, which functions as a binding site for the Nuclear Factor Y (NF-Y) complex, a key player in plant heat stress responses [49]. The expression of *QT12* is inversely correlated with grain quality under heat stress conditions. Elevated *QT12* expression disrupts the homeostasis of endosperm storage materials, resulting in increased amylose content and decreased protein content, thereby leading to enhanced grain chalkiness. Conversely, regarding *QT12*, low expression or loss-of-function alleles promote the maintenance of storage substance balance and improve grain quality. In extensive high-temperature field trials, reduced *QT12* expression significantly enhanced the seed setting rate and per-plant yield of rice while also improving the eating quality [8].

### 3.4. HTS1 and Regulation of Membrane Homeostasis

Membrane lipid homeostasis is a pivotal factor determining plant thermotolerance, as elevated temperatures can modify lipid composition, compromise membrane integrity, and induce the overaccumulation of reactive oxygen species (ROS).

*OsEDS1* acts as a positive regulator of heat tolerance in rice by maintaining hydrogen peroxide (H_2_O_2_) homeostasis. Loss-of-function *oseds1* mutants demonstrate significant heat sensitivity, with a survival rate of merely 33.6% compared to 73.4% in wild-type (WT) plants. These mutants also exhibit increased ion leakage and diminished chlorophyll content under heat stress conditions. Complementation lines successfully restore thermotolerance to WT levels, and mutational analysis indicates that the conserved S143 residue is not essential in the functional activity of *OsEDS1.*

An additional upstream regulator, *HTS1* (Heat Tolerance Sensitive 1), was identified through EMS mutagenesis screening and subsequently cloned using map-based cloning techniques. *HTS1* encodes a β-ketoacyl carrier protein reductase (KAR) localized to the chloroplast thylakoid membrane, which serves as a crucial enzyme in fatty acid biosynthesis. Under high-temperature stress, *hts1* mutants exhibit a reduction in membrane lipid unsaturation, leading to excessive ROS accumulation and enhanced programmed cell death (PCD). These perturbations compromise the stability of membrane systems and disrupt heat signal transduction, highlighting *HTS1*’s role as an upstream core regulator in the rice heat response pathway [18]. *OsEDS1* and *HTS1* together exemplify the intricate coordination between reactive oxygen species (ROS) homeostasis and membrane lipid composition, forming a functional module that is crucial in maintaining cellular integrity and initiating effective heat stress signaling in rice.

### 3.5. Antioxidant-Related Regulators in the OsHTAS Network

In addition to *OsHTAS* (discussed in detail in Section 2.5), several antioxidant-related regulators act synergistically to reinforce thermotolerance in rice. Several key regulators interact synergistically with *OsHTAS* to establish a complex thermotolerance network. *PSL50* acts as a positive regulator by mitigating reactive oxygen species (ROS) accumulation and reducing cell death under heat stress conditions. Mutants lacking *PSL50* exhibit lower survival rates, although this phenotype is partially ameliorated under high-light conditions. *OsProDH* serves as a negative regulator of thermotolerance; its gene knockout results in enhanced survival under heat stress, whereas its overexpression diminishes tolerance. *OsMDHAR4* operates as a negative regulator of hydrogen peroxide-induced stomatal closure, with knockout mutants demonstrating improved survival rates. *OsMYB55* plays a role in enhancing amino acid metabolism, thereby contributing to thermotolerance; lines overexpressing this gene experience less yield loss under elevated temperature conditions. Together, these genes cooperate with *OsHTAS* to maintain redox equilibrium and minimize oxidative damage during heat stress.

### 3.6. SLG1 and tRNA Thiolation

*SLG1* (Slender Guy 1) encodes the cytoplasmic tRNA 2-thiolation protein 2 (RCTU2). Loss-of-function mutants (*slg1*) show significantly reduced levels of 2-thiolation at tRNA sites, leading to heat-sensitive phenotypes during both the seedling and reproductive stages in rice. In contrast, *SLG1* overexpression elevates tRNA thiolation levels and enhances heat tolerance. Notably, allelic variation in *SLG1* is observed between the indica and japonica subspecies, exemplified by *Hap2* versus *Hap1*. Rice possessing the indica allele demonstrates enhanced heat tolerance, attributable to natural variations in both the promoter and coding regions, which contribute to this phenotypic distinction [37].

### 3.7. Other Key Genes

Through forward genetic screening of the Taiwan Rice Insertional Mutant (TRIM) library, the mutant line M35973 was identified, characterized by significantly enlarged grains. This phenotype resulted from the T-DNA activation of *LOC_Os11g30430 (RBG1)*, located 2.6 kb upstream of the insertion site. Functional analyses revealed that *RBG1* enhances cell division and organ development, suggesting that the targeted enhancement of this gene could simultaneously improve yield-related traits and thermotolerance-associated growth resilience [50].

## 4. Mechanisms of Crosstalk Between Rice Heat Tolerance and Grain Development

### 4.1. Long-Distance ABA Transport and Grain Filling

Effective grain filling under elevated temperature conditions is crucial in sustaining rice yield and quality. Among the regulatory mechanisms involved, the long-distance transport of abscisic acid (ABA) from source to sink tissues is critical during panicle grain filling. ABA synthesized in the leaves is translocated via the MATE family transporter DG1 from stem nodes to developing grains, where it upregulates the expression of starch synthesis genes and stabilizes grain filling under heat stress [5,51]. Under prolonged exposure to 38 °C, *dg1* mutants exhibit significantly impaired grain filling, a marked reduction in 1000-grain weight, and an increased proportion of shriveled grains. The seed setting rate of wild-type plants under these conditions reaches 68.5%, whereas that of *dg1* mutants decreases to 42.3% (*p* < 0.01). Heat stress strongly induces DG1 expression, enhancing ABA transport efficiency and mitigating heat-induced defects in grain development. This demonstrates that DG1 is a promising molecular target for breeding heat-tolerant rice varieties.

In addition to the regulation mediated by abscisic acid (ABA), several genetic factors play a crucial role in enhancing heat resilience during the grain-filling stage. The *FLO2* gene is particularly noteworthy; loss-of-function mutants of *flo2* exhibit an 11% reduction in grain weight and alterations in starch granule structure. Interestingly, the transcriptional response of *FLO2* to heat stress varies between cultivars; it is upregulated in Kinmaze but downregulated in *Nipponbare*, which suggests genotype-specific regulatory mechanisms [31]. The *Sus3* and *Apql* genes also contribute significantly; the *Sus3Haba* allele reduces the occurrence of white-back and white-belly grains under heat stress, with both the promoter and coding regions influencing this phenotype [32]. The *Apql-NIL* line provides tolerance specifically during the early grain-filling phase, occurring 3–5 days after flowering (DAF). The TAP gene is another critical factor; *tap* mutants exhibit panicle defects at temperatures equal to or exceeding 30 °C, yet they remain phenotypically normal at 22 °C, underscoring the temperature sensitivity of reproductive development [52]. Furthermore, the overexpression of the *AtpB* gene enhances grain integrity and reduces chalkiness under high-temperature stress [33]. Lastly, mutations in *OsDML4* lead to the hypermethylation of the *RISBZ1* promoter, which suppresses the synthesis of storage proteins and increases grain chalkiness [34]. Elevated temperatures induce the expression of the β isoform of *OsbZIP58*, characterized by diminished transcriptional activity, thereby resulting in a reduced accumulation of starch and proteins. Japonica rice cultivars exhibit heightened sensitivity due to a greater prevalence of the β isoform [35]. Regarding *FLO11-2* (*cpHSP70-2*), the *D259V* mutation diminishes ATPase activity, contributing to increased grain chalkiness under heat stress [36]. The gene *cpHSP70-2* is highly expressed in the endosperm, indicating its potential role in protein folding during grain development.

### 4.2. Regulation of Endosperm Development by MADS-Box Transcription Factors

Members of the MADS-box family, including *OsMADS87*, are integral in rice endosperm development and exhibit high sensitivity to elevated temperatures. Heat stress markedly suppresses *OsMADS87* expression, a suppression further exacerbated by promoter methylation, resulting in delayed endosperm cellularization and reduced grain size [53]. Specifically, exposure to 35 °C results in a reduction of approximately 60% in *OsMADS87* expression, an increase in promoter DNA methylation, and a delay in endosperm cellularization. Functional analyses reveal that *OsMADS87* overexpression lines experience a 15.3% increase in grain length under heat stress, whereas RNAi lines exhibit an 18.7% decrease. These findings suggest that *OsMADS87* influences grain size by regulating endosperm cell differentiation, and its sensitivity to heat stress elucidates a molecular mechanism underlying heat-induced developmental imbalances in grains.

### 4.3. Impact of Heat Stress During the Grain-Filling Window

Rice exhibits developmental stage-specific sensitivity to elevated temperatures, with the grain-filling stage—particularly 15 to 25 days post-heading—emerging as the phase most susceptible to heat stress. Controlled-environment studies have demonstrated that exposure to temperatures exceeding 35 °C for a duration as brief as six hours during this critical period can lead to a reduction in grain plumpness of approximately 28.5% and an increase in chalkiness of 42.7%, culminating in significant yield loss and quality degradation [5,54]. At the molecular level, heat stress disrupts the coordinated regulation of abscisic acid (ABA) signaling and starch biosynthesis genes, such as *GBSSI* and *SSIIa*, thereby impairing starch deposition and compromising grain filling [5,55,56].

**Figure 2:**
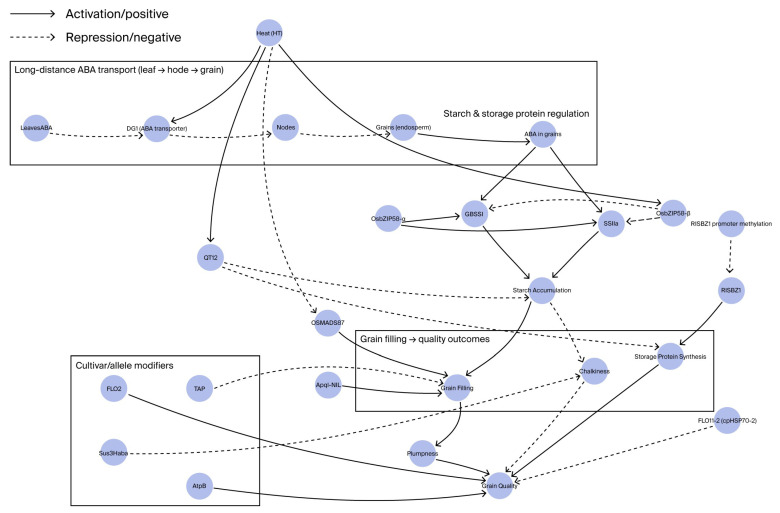
A schematic model detailing the long-distance ABA transport mediated by DG1 and its regulation of grain filling under heat stress, alongside the influence of other key genetic modules (see Section 4.1 for full details).

These reports suggest that the grain-filling stage represents a critical juncture where environmental stress intersects with multiple genetic and epigenetic pathways governing starch synthesis, protein accumulation, and grain quality. Breeding strategies that target these genes, in conjunction with optimized ABA transport and signaling, present promising approaches to enhancing heat resilience during this crucial yield-determining phase. A schematic diagram illustrating the role of abscisic acid (ABA) in long-distance signaling and its integration with starch and protein biosynthesis under heat stress is shown in Figure 2.

*FLO2* influences endosperm development and grain weight, exhibiting heat-response variability among different cultivars. The *Sus3Haba* allele and *Apql-NIL* lines enhance grain appearance by reducing the ratios of white-back and white-belly grains, particularly during the critical 3–5 days after flowering (DAF) in the grain-filling period. *Tap* mutants display panicle defects at temperatures of 30 °C or higher. The overexpression of AtpB improves grain integrity under elevated temperatures, while mutations in *FLO11-2* (*cpHSP70-2*) impair ATPase activity, leading to increased chalkiness. The repression and promoter hypermethylation of *OsMADS87* delay endosperm cellularization, adversely affecting grain size and quality.

## 5. Emerging Technologies for Rice Heat Tolerance Research

### 5.1. Multi-Omics Approaches in Heat Tolerance Research

The integration of multi-omics approaches—including genomics, transcriptomics, proteomics, metabolomics, and epigenomics—has become essential in elucidating the molecular mechanisms underlying heat tolerance in rice. These methodologies enable comprehensive insights into gene regulation, metabolite dynamics, and epigenetic modifications under heat stress, thereby identifying candidate genes, pathways, and markers for breeding programs.

Epigenomic studies further highlight the role of DNA methylation and histone modifications, exemplified by the dynamic remodeling of *H2A.Z* nucleosomes and the regulation of heat-responsive genes [43]. Similarly, combined transcriptome–metabolome analysis in Dongxiang wild rice demonstrated the activation of antioxidant pathways and sugar metabolism, accompanied by the accumulation of citrate and malate and the upregulation of *HSPs* [57]. Notably, heat-tolerant varieties often exhibit the promoter demethylation of stress-response genes, whereas heat-sensitive varieties display increased methylation that suppresses gene expression [57]. These epigenetic patterns suggest that promoter demethylation could serve as a selectable marker in breeding programs, where stable epialleles associated with enhanced thermotolerance may be utilized to improve rice resilience under heat stress.

### 5.2. Applications of Gene Editing

Gene-editing technologies, particularly *CRISPR/Cas9*, hold significant potential for enhancing heat tolerance in rice. By precisely targeting and modifying heat-related genes, it is possible to improve physiological traits and stabilize yields under elevated temperatures [58]. For instance, Zhang et al. [59] in 2016 utilized *CRISPR/Cas9* to edit the heat shock element (HSE) region within the *OsHSP16.9* promoter, resulting in enhanced heat-induced gene expression. The edited rice lines demonstrated accelerated starch accumulation and maintained yield stability under stress conditions of 38 °C during the grain-filling stage, without exhibiting any visible growth defects [59]. In another study, Liu et al. [22] employed *CRISPR/Cas9* to generate *OsNTL3*-knockout mutants, which displayed heightened sensitivity to heat at 45 °C. Conversely, ΔTM lines, which lack membrane-anchoring domains, exhibited increased heat tolerance, demonstrating that *OsNTL3* is a crucial regulatory factor [22]. The functional validation of candidate genes such as *OsCNGC14/16* (calcium channels) and *HTG3* (heat shock factors) through knockout or domain deletion provides a rapid assessment of their roles in heat response, offering valuable targets for breeding programs [20,58]. Beyond the widely applied CRISPR/Cas9 system, recent genome-editing innovations such as CRISPR/Cas12a (Cpf1), prime editing, and base editing have greatly expanded the genetic toolbox for rice improvement [60,61,62].

## 6. Molecular Breeding Strategies and Integration of Technology

### 6.1. Marker-Assisted Selection (MAS)

Marker-assisted selection (MAS) represents an effective breeding strategy in enhancing heat tolerance in rice. The integration of known heat-tolerant quantitative trait loci (QTLs) into elite cultivars can significantly improve spikelet fertility and yield stability under elevated temperature conditions. For instance, QTLs such as *qHTSF1.1* and *qHTSF4.1* have been validated in the *IR64* × *N22* cross, with *qHTSF4.1* demonstrating an increase in spikelet fertility of approximately 17.6% [11]. Additionally, natural alleles from African rice, including *TT1* and *SLG1*, have been successfully introduced into Asian rice backgrounds, resulting in substantial improvements in heat tolerance [57]. Considering that most QTLs exhibit minor effects (R^2^ < 5%), it is recommended that MAS employ gene pyramiding strategies by stacking QTLs such as *qHTSF1.1*, *qHTSF4.1*, *TT1*, and *SLG1*. This approach is designed to concurrently enhance multiple heat-resistance pathways, including membrane stability, water regulation, and protein homeostasis [63].

### 6.2. Gene Editing in Heat Tolerance Breeding

The *CRISPR/Cas9* system provides precise tools for enhancing gene function under heat stress without compromising growth. Liu et al. [22] demonstrated the potential of *OsNTL3* editing by developing ΔTM mutants that exhibited significantly improved heat tolerance at 45 °C, thereby confirming their breeding potential [22]. In a subsequent study, Liu et al. [64] utilized CRISPR to knock out *OsRbohB*, resulting in reduced reactive oxygen species (ROS) accumulation and an increase in yield of 8.9–20.5% under heat stress conditions [64]. Although *OsHTAS* was not directly edited, its role in ROS scavenging and stomatal regulation, along with *OsAPX8*, suggests promising targets for future *CRISPR* applications [19].

### 6.3. Gene Pyramiding and Breeding by Design

The strategy of gene pyramiding, which combines multiple heat tolerance genes, offers synergistic improvements. The integration of genome-wide association studies (GWASs) and genomic selection (GS) facilitates the prediction of multi-gene effects under heat stress. Li et al. [65] identified heat-related quantitative trait loci (QTLs) such as *qHT7* and *qHTT4.2*, as well as genes such as *OsVQ30*, through GWASs and confirmed these findings using transcriptomics [65,66]. Studies on GS have demonstrated its efficacy in improving the integration of small-effect QTLs. For instance, Grenier et al. [67] applied GS to predict flowering time, plant height, and yield in upland rice, achieving a genomic estimated breeding value (GEBV) accuracy ranging from 0.12 to 0.54 [67]. G×E interactions have been extensively studied, with research such as that by Zhang et al. [68] demonstrating that the integration of genome-wide association study (GWAS) single-nucleotide polymorphisms (SNPs) into genomic selection (GS) models enhances the accuracy of heat tolerance prediction to 0.66 [68]. The pyramiding of genes such as *TT1*, *SLG1*, *TT2*, and *TT3* has yielded field-validated results, indicating yield advantages under heat stress conditions [12,69].

### 6.4. Coordinated Improvement of Multiple Traits

Breeding for heat tolerance often involves trade-offs with other agronomic traits. For instance, the overexpression of *OsDREB2A* has been shown to enhance heat tolerance but results in a reduction in tiller number by approximately 20% [21,70]. Contemporary strategies to address these challenges include 1) tissue-specific expression, utilizing pollen- or flowering-specific promoters, such as *HSFA2*, to enhance heat tolerance without negatively impacting vegetative growth [71] and 2) a combination of heat tolerance and yield-related genes, where stacking *TT3* with *GS3* can alleviate antagonistic effects and concurrently improve both heat tolerance and yield [13,72].

## 7. Discussion

Global warming and increased heat events represent escalating challenges in rice production. Empirical studies indicate that a 1 °C rise in temperature can lead to an approximate 3.85% reduction in rice yield, particularly during the flowering and grain-filling stages [73]. This reduction is attributed to heightened sterility, decreased 1000-grain weight, and deterioration in starch quality [74]. Recent reviews spanning from 2023 to 2025 demonstrate significant advancements in understanding the mechanisms behind heat tolerance in rice and the development of breeding strategies. However, there are four primary obstacles that persist: fragmented regulatory models, disconnection between laboratory simulations and field conditions, inadequate functional and phenotypic validation in field conditions, and insufficient integration of traits [7,13,17,58]. To overcome these challenges, systematic breakthroughs are required in both fundamental research and applied breeding.

### 7.1. Fragmented Mechanistic Models

Although numerous heat-response modules have been identified, they remain isolated. A comprehensive network that encompasses membrane signaling, nuclear transcription, RNA stabilization, and physiological responses is absent. For instance, *HTS1* encodes a chloroplast membrane enzyme that regulates reactive oxygen species and *HSFA2* activity, highlighting its role as an initiator of membrane signaling [18]. Additionally, the ALBA–HSF mRNA–phase separation axis is crucial, yet its connection to membrane or epigenetic signals remains unclear [75].

### 7.2. Disconnect Between Laboratory Simulations and Field Conditions

Laboratory studies frequently employ acute heat treatments (e.g., 42 °C for 2–6 h) to identify acute-response genes such as *HSP101* or *HSFA2d*. However, these conditions differ significantly from chronic stress experienced in field environments (35–38 °C for 5–10+ days), which limits the practical applicability of the candidate genes identified. Notably, nighttime heat can have a profound impact on grain filling [5]. Emerging long-term simulation platforms, such as heat tents that increase nighttime temperatures by +3–4 °C, are beginning to address this discrepancy by more accurately reflecting field conditions.

### 7.3. Functional Validation and Breeding Translation Lag Behind

Although numerous candidate genes (e.g., *qHST1*, *qHTT8*, *TT3.1*, and *OsMADS87*) have been identified, comprehensive functional validation remains limited. For example, while qHTT8 has been mapped using BSA-seq, the downstream mechanisms involving *OsNAC31* are still not fully understood [65]. Techniques such as CRISPR have facilitated the validation of genes such as *OsNTL3*, *OsNAC006*, and *OsRbohB*. However, further research is necessary to assess their stability across different growth stages and environmental conditions [64].

### 7.4. Trait Trade-Offs Limit Breeding Efficiency

Enhancing heat tolerance in rice often results in trade-offs with yield or quality. For instance, DG1 plays a role in regulating ABA transport and grain filling, yet it influences starch structure, while *TT3* increases heat tolerance but can potentially lower the 1000-grain weight in certain lines [30,65]. In addition, significant challenges are encountered in aggregating multiple superior genes into the same superior genetic background (variety).

### 7.5. Limitations of This Review

Climate warming leads not only to higher temperatures but also to increased carbon dioxide concentrations. Although high-temperature heat damage can severely affect rice yields, studies have shown that an increase in carbon dioxide concentration can enhance rice production by 7.1% [73]. In this study, only the genetic and molecular basis of rice heat tolerance was considered, without taking into account the interaction effects and mechanisms regarding the increase in carbon dioxide concentration. In the future, further research should be carried out on the genetic and molecular regulatory mechanisms of rice under multiple environmental influences.

## 8. Conclusions

Research on rice heat tolerance must transition from the discovery of genes to their integration into, and application in, breeding programs. Future endeavors should prioritize demand-driven strategies, underpinned by multi-omics approaches, precision genome editing, and advanced breeding techniques, to develop “designable, quantifiable, and scalable” heat-resilient rice varieties, with the aim of providing a viable solution to the challenges posed by climate change in global rice production.

## Figures and Tables

**Table 1:** Functional classification and mechanisms of rice heat tolerance-related genes with references.

Category	Gene/Locus	Functional Description	Regulatory Mechanism/Pathway	Heat Tolerance Effect
Heat Sensing and Signal Transduction	*HTS1* [18]	β-ketoacyl carrier protein reductase in chloroplast thylakoid membrane regulates lipid unsaturation	Maintains membrane stability, suppresses ROS accumulation, and modulates Ca^2+^ signaling	Loss leads to membrane rupture, increased PCD; normal function enhances heat perception
	*OsHTAS* [19]	RING-type E3 ubiquitin ligase	Regulates H_2_O_2_ accumulation to promote stomatal closure; interacts with *OsAPX8* for ROS scavenging	Overexpression markedly improves survival under heat stress
	*TT2* [15]	G protein γ subunit	Regulates Ca^2+^–CaM–SCT1–OsWR2 pathway to maintain wax deposition	Preserves leaf surface barrier and improves heat tolerance
Transcriptional Regulation	*OsHSFA2e/* [20]	Heat shock transcription factor; binds HSE to activate stress genes	Induces HSPs (*OsHSP70*, *OsHSP90*) and activates *OsHsfA2d* and stress TFs (*OsSNAC1*, *OsDREB2A*, *OsLEA3*)	Enhances floral organ and whole-plant thermotolerance
	*OsHSFA2d* [21]	Core Hsf in heat response	Upregulates HSPs and LEAs and stabilizes proteins and membranes	Improves survival and reproductive stability under heat
	*OsNTL3–OsbZIP74* [22]	Membrane-tethered NAC TF (*OsNTL3*) and spliced bZIP TF (*OsbZIP74*)	Heat/ER stress → *OsNTL3* cleavage and nuclear import. *OsbZIP74* activates *OsNTL3*; both form a positive loop to induce UPR/heat-responsive genes	Enhances stress gene expression, reduces ROS, and improves survival under heat
	*OsDOF27* [23]	Plant-specific DOF transcription factor with intrinsically disordered protein (IDP) features; is nuclear-localized	Heat-inducible; promoter enriched in HSE/ABRE motifs. May self-regulate via DOF sites; activates *HSPs* and stress-related genes	Overexpression lines show ~2× survival rate
	*WRKY10–VQ8* [24]	*WRKY–VQ* interaction module	Suppresses pro-senescence genes and reduces ROS accumulation	Delays cell death and improves survival under heat stress
	*OsMYB55* [25]	*R2R3-MYB* transcription factor	*R2R3-MYB* transcription factor that promotes amino acid biosynthesis and maintains metabolic stability	Reduces yield loss under high temperatures
Protein Homeostasis and Degradation	*TT1* [14]	26S proteasome α2 subunit	Degrades heat-damaged proteins and maintains proteostasis	The elite allele improves heat adaptation significantly
	*TT3.1–TT3.2* [9]	E3 ubiquitin ligase–chloroplast chaperone	Promotes *TT3.2* degradation and protects PSII	Enhances photosynthesis and yield under heat
Membrane Lipid and Antioxidant Regulation	*OsEDS1* [26]	Positive regulator of thermotolerance; interacts with catalases	Stabilizes and enhances the activity of *OsCATB/OsCATC* in peroxisomes; maintains H_2_O_2_ homeostasis under heat	Overexpression reduces ROS accumulation and improves survival, fertility, grain weight, and yield under heat
	*PSL50* [27]	Premature senescence-related protein	Reduces ROS accumulation and cell death	Mutants display decreased thermotolerance
	*OsProDH* [28]	Proline dehydrogenase catalyzes proline degradation to P5C	Promotes proline catabolism; knockout increases proline accumulation, and overexpression decreases proline	Negative regulator of thermotolerance: knockout reduces ROS and enhances survival; overexpression increases ROS and sensitivity under heat
	*OsMDHAR4* [29]	Ascorbate metabolism enzyme	Inhibits H_2_O_2_-induced stomatal closure	Knockout enhances survival
Grain Development and Quality-Related Genes	*QT12* [8]	Grain quality regulator; expression negatively correlated with thermotolerance	NF-YA8–NF-YB9/NF-YC10 module controls *QT12* via CCAAT-box. Indica promoter variation disrupts NF-YA8 binding → low *QT12* expression; japonica retains binding → high expression	Low *QT12* expression maintains starch–protein balance and improves fertility, yield, and grain quality under heat; high *QT12* expression increases chalkiness and reduces tolerance
	*DG1* [30]	MATE transporter essential for seed filling; mediates ABA efflux	Promotes ABA long-distance transport and stabilizes grain filling	Maintains grain filling and starch synthesis under heat
	*FLO2* [31]	Participates in the regulation of grain development under high-temperature stress	Affects grain filling and endosperm development	Improves grain shape, quality, and yield under heat
	*Sus3Haba* [32]	Maintains starch synthesis and reduces the formation of chalky grains		
	*Apql* [32]	Heat response sucrose synthase Sus3		
	*AtpB* [33]	Responsible for driving ATP synthesis and playing a crucial role in energy supply and grain development		
	*OsDML4* [34]	Maintains a genome-wide hypomethylation state at high temperatures		
	*OsbZIP58* [35]	Maintains the normal development of grains by activating starch and storage protein synthesis genes to inhibit the expression of hydrolase genes		
	*FLO11-2* [36]	Regulate rice grain quality under high temperatures		
tRNA Modification	*SLG1* [37]	Is a member of the CTU2 superfamily; regulates tRNA 2-thiolation; and is localized in the nucleus and cytoplasm	Heat-inducible; controls tRNA 2-thiolation levels; loss reduces thiolation and thermotolerance; and overexpression enhances them	Knockout causes heat sensitivity; indica haplotype (*Hap2*) confers stronger tolerance than japonica (*Hap1*)

## Data Availability

Not mentioned.

## References

[1] Intergovernmental Panel on Climate Change (2021). Climate Change 2021: The Physical Science Basis. Contribution of Working Group I to the Sixth Assessment Report of the Intergovernmental Panel on Climate Change.

[2] Ju Y., Choi J., Yun S., Mittra P.K., Woo S., Sakagami J.I. (2025). Stage-dependent heat priming mitigates reproductive heat stress via proteomic regulation in *Oryza sativa* L.. Plant Sci..

[3] Prerostova S., Jarošová J., Dobrev P., Gaudinova A., Knirsch V., Kobzova E., Benczúr K., Szalai G., Novak O., Vankova R. (2025). Cytokinin elevation caused by high light intensity contributes substantially to the increase of thermotolerance of rice plants. Plant Stress.

[4] Kompas T., Che T.N., Grafton R.Q. (2024). Global impacts of heat and water stress on food production and severe food insecurity. Sci. Rep..

[5] Lu F., Feng B., Chen L., Qiu J., Wei X. (2025). How Does Rice Cope with High-Temperature Stress During Its Growth and Development, Especially at the Grain-Filling Stage?. Agronomy.

[6] Park J.R., Kim E.G., Jang Y.H., Kim K.M. (2021). Screening and identification of genes affecting grain quality and spikelet fertility during high-temperature treatment in grain filling stage of rice. BMC Plant Biol..

[7] Riaz A., Thomas J., Ali H.H., Zaheer M.S., Ahmad N., Pereira A. (2024). High night temperature stress on rice (*Oryza sativa*)—insights from phenomics to physiology. A review. Funct. Plant Biol..

[8] Li W., Yang K., Hu C., Abbas W., Zhang J., Xu P., Cheng B., Zhang J., Yin W., Shalmani A. (2025). A natural gene on-off system confers field thermotolerance for grain quality and yield in rice. Cell.

[9] Zhang H., Zhou J.-F., Kan Y., Shan J.-X., Ye W.-W., Dong N.-Q., Guo T., Xiang Y.-H., Yang Y.-B., Li Y.-C. (2022). A genetic module at one locus in rice protects chloroplasts to enhance thermotolerance. Science.

[10] Liu X., Zhong X., Liao J., Ji P., Yang J., Cao Z., Duan X., Xiong J., Wang Y., Xu C. (2023). Exogenous abscisic acid improves grain filling capacity under heat stress by enhancing antioxidative defense capability in rice. BMC Plant Biol..

[11] Liu H., Zeng B., Zhao J., Yan S., Wan J., Cao Z. (2023). Genetic Research Progress: Heat Tolerance in Rice. Int. J. Mol. Sci..

[12] Xing Y.H., Lu H., Zhu X., Deng Y., Xie Y., Luo Q., Yu J. (2024). How Rice Responds to Temperature Changes and Defeats Heat Stress. Rice.

[13] Liu H., Wei Y., Xia S., Xie W., Ren D., Rao Y. (2025). Improvements in Tolerance to Heat Stress in Rice via Molecular Mechanisms and Rice Varieties. Agriculture.

[14] Li X.M., Chao D.Y., Wu Y., Huang X., Chen K., Cui L.G., Su L., Ye W.W., Chen H., Chen H.C. (2015). Natural alleles of a proteasome α2 subunit gene contribute to thermotolerance and adaptation of African rice. Nat. Genet..

[15] Kan Y., Mu X.R., Zhang H., Gao J., Shan J.X., Ye W.W., Lin H.X. (2022). TT2 controls rice thermotolerance through SCT1-dependent alteration of wax biosynthesis. Nat. Plants.

[16] Ps S., Sv A.M., Prakash C., Mk R., Tiwari R., Mohapatra T., Singh N.K. (2017). High Resolution Mapping of QTLs for Heat Tolerance in Rice Using a 5K SNP Array. Rice.

[17] Yiwei F., Jiayelu W., Mingming W., Shenghai Y., Rongrong Z., Jing Y., Guofu Z., Faming Y., Yanting L., Xiaoming Z. (2024). Progress on Molecular Mechanism of Heat Tolerance in Rice. Rice Sci..

[18] Chen F., Dong G., Wang F., Shi Y., Zhu J., Zhang Y., Ruan B., Wu Y., Feng X., Zhao C. (2021). A β-ketoacyl carrier protein reductase confers heat tolerance via the regulation of fatty acid biosynthesis and stress signaling in rice. New Phytol..

[19] Liu J., Zhang C., Wei C., Liu X., Wang M., Yu F., Xie Q., Tu J. (2016). The RING Finger Ubiquitin E3 Ligase OsHTAS Enhances Heat Tolerance by Promoting H_2_O_2_-Induced Stomatal Closure in Rice. Plant Physiol..

[20] Wu N., Yao Y., Xiang D., Du H., Geng Z., Yang W., Li X., Xie T., Dong F., Xiong L. (2022). A MITE variation-associated heat-inducible isoform of a heat-shock factor confers heat tolerance through regulation of JASMONATE ZIM-DOMAIN genes in rice. New Phytol..

[21] Mei W., Chen W., Wang Y., Liu Z., Dong Y., Zhang G., Deng H., Liu X., Lu X., Wang F. (2023). Exogenous Kinetin Modulates ROS Homeostasis to Affect Heat Tolerance in Rice Seedlings. Int. J. Mol. Sci..

[22] Liu X.H., Lyu Y.S., Yang W., Yang Z.T., Lu S.J., Liu J.X. (2020). A membrane-associated NAC transcription factor OsNTL3 is involved in thermotolerance in rice. Plant Biotechnol. J..

[23] Gandass N., Kajal, Salvi P. (2022). Intrinsically disordered protein, DNA binding with one finger transcription factor (OsDOF27) implicates thermotolerance in yeast and rice. Front. Plant Sci..

[24] Chen S., Cao H., Huang B., Zheng X., Liang K., Wang G.L., Sun X. (2022). The WRKY10-VQ8 module safely and effectively regulates rice thermotolerance. Plant Cell Environ..

[25] El-Kereamy A., Bi Y.M., Ranathunge K., Beatty P.H., Good A.G., Rothstein S.J. (2012). The rice R2R3-MYB transcription factor OsMYB55 is involved in the tolerance to high temperature and modulates amino acid metabolism. PLoS ONE.

[26] Liao M., Ma Z., Kang Y., Zhang B., Gao X., Yu F., Yang P., Ke Y. (2023). ENHANCED DISEASE SUSCEPTIBILITY 1 promotes hydrogen peroxide scavenging to enhance rice thermotolerance. Plant Physiol..

[27] He Y., Zhang X., Shi Y., Xu X., Li L., Wu J.L. (2021). PREMATURE SENESCENCE LEAF 50 Promotes Heat Stress Tolerance in Rice (*Oryza sativa* L.). Rice.

[28] Guo M., Zhang X., Liu J., Hou L., Liu H., Zhao X. (2020). OsProDH Negatively Regulates Thermotolerance in Rice by Modulating Proline Metabolism and Reactive Oxygen Species Scavenging. Rice.

[29] Liu J., Sun X., Xu F., Zhang Y., Zhang Q., Miao R., Zhang J., Liang J., Xu W. (2018). Suppression of OsMDHAR4 enhances heat tolerance by mediating H_2_O_2_-induced stomatal closure in rice plants. Rice.

[30] Qin P., Zhang G., Hu B., Wu J., Chen W., Ren Z., Liu Y., Xie J., Yuan H., Tu B. (2021). Leaf-derived ABA regulates rice seed development via a transporter-mediated and temperature-sensitive mechanism. Sci. Adv..

[31] She K.C., Kusano H., Koizumi K., Yamakawa H., Hakata M., Imamura T., Fukuda M., Naito N., Tsurumaki Y., Yaeshima M. (2010). A novel factor FLOURY ENDOSPERM2 is involved in regulation of rice grain size and starch quality. Plant Cell.

[32] Takehara K., Murata K., Yamaguchi T., Yamaguchi K., Chaya G., Kido S., Iwasaki Y., Ogiwara H., Ebitani T., Miura K. (2018). Thermo-responsive allele of sucrose synthase 3 (Sus3) provides high-temperature tolerance during the ripening stage in rice (*Oryza sativa* L.). Breed. Sci..

[33] Kusano H., Arisu Y., Nakajima J., Yaeshima M., She K.-C., Shimada H. (2016). Implications of the gene for F1–ATPase β subunit (AtpB) for the grain quality of rice matured in a high-temperature environment. Plant Biotechnol..

[34] Yan Y., Li C., Liu Z., Zhuang J.J., Kong J.R., Yang Z.K., Yu J., Shah Alam M., Ruan C.C., Zhang H.M. (2022). A new demethylase gene, OsDML4, is involved in high temperature-increased grain chalkiness in rice. J. Exp. Bot..

[35] Xu H., Li X., Zhang H., Wang L., Zhu Z., Gao J., Li C., Zhu Y. (2020). High temperature inhibits the accumulation of storage materials by inducing alternative splicing of OsbZIP58 during filling stage in rice. Plant Cell Environ..

[36] Tabassum R., Dosaka T., Ichida H., Morita R., Ding Y., Abe T., Katsube-Tanaka T. (2020). FLOURY ENDOSPERM11-2 encodes plastid HSP70-2 involved with the temperature-dependent chalkiness of rice (*Oryza sativa* L.) grains. Plant J..

[37] Xu Y., Zhang L., Ou S., Wang R., Wang Y., Chu C., Yao S. (2020). Natural variations of SLG1 confer high-temperature tolerance in indica rice. Nat. Commun..

[38] Yokotani N., Ichikawa T., Kondou Y., Matsui M., Hirochika H., Iwabuchi M., Oda K. (2008). Expression of rice heat stress transcription factor OsHsfA2e enhances tolerance to environmental stresses in transgenic Arabidopsis. Planta.

[39] Rao S., Gupta A., Bansal C., Sorin C., Crespi M., Mathur S. (2022). A conserved HSF:miR169:NF-YA loop involved in tomato and Arabidopsis heat stress tolerance. Plant J..

[40] Kotak S., Larkindale J., Lee U., von Koskull-Doring P., Vierling E., Scharf K.D. (2007). Complexity of the heat stress response in plants. Curr. Opin. Plant Biol..

[41] Schramm F., Ganguli A., Kiehlmann E., Englich G., Walch D., von Koskull-Doring P. (2006). The heat stress transcription factor HsfA2 serves as a regulatory amplifier of a subset of genes in the heat stress response in Arabidopsis. Plant Mol. Biol..

[42] Jin Q., Chachar M., Ali A., Chachar Z., Zhang P., Riaz A., Ahmed N., Chachar S. (2024). Epigenetic Regulation for Heat Stress Adaptation in Plants: New Horizons for Crop Improvement under Climate Change. Agronomy.

[43] Cortijo S., Charoensawan V., Brestovitsky A., Buning R., Ravarani C., Rhodes D., van Noort J., Jaeger K.E., Wigge P.A. (2017). Transcriptional Regulation of the Ambient Temperature Response by H2A.Z Nucleosomes and HSF1 Transcription Factors in Arabidopsis. Mol. Plant.

[44] Huang Y., An J., Sircar S., Bergis C., Lopes C.D., He X., Da Costa B., Tan F.Q., Bazin J., Antunez-Sanchez J. (2023). HSFA1a modulates plant heat stress responses and alters the 3D chromatin organization of enhancer-promoter interactions. Nat. Commun..

[45] Zheng X., Zuo Z., Yao P., Li X., Zhang Q., Chen X. (2025). Bromodomain-containing proteins interact with a non-canonical RNA polymerase II kinase to maintain gene expression upon heat stress. Nat. Plants.

[46] Biswas S., Islam M.N., Sarker S., Tuteja N., Seraj Z.I. (2019). Overexpression of heterotrimeric G protein beta subunit gene (OsRGB1) confers both heat and salinity stress tolerance in rice. Plant Physiol. Biochem..

[47] Liu J., Wang K., Wang G., Peng Z., Wang T., Meng Y., Huang J., Huo J., Li X., Zhu X. (2025). The OsEBF1-OsEIL5-OsPP91 module regulates rice heat tolerance via ubiquitination and transcriptional activation. Cell Rep..

[48] Ma Y., Zhao S., Ma X., Dong G., Liu C., Ding Y., Hou B. (2025). A high temperature responsive UDP-glucosyltransferase gene OsUGT72F1 enhances heat tolerance in rice and Arabidopsis. Plant Cell Rep..

[49] Yang B., Xie Y., Liu Y. (2025). A novel NF-Ys-QT12-IRE1 module controlling grain quality and yield thermotolerance in rice. Mol. Plant.

[50] Lo S.F., Cheng M.L., Hsing Y.C., Chen Y.S., Lee K.W., Hong Y.F., Hsiao Y., Hsiao A.S., Chen P.J., Wong L.I. (2020). Rice Big Grain 1 promotes cell division to enhance organ development, stress tolerance and grain yield. Plant Biotechnol. J..

[51] Xiong J., Wang H., Zhong Z., Li S., Qin P. (2025). Emerging strategies to improve heat stress tolerance in crops. aBIOTECH.

[52] Zhang P., Zhu W., He Y., Fan J., Shi J., Fu R., Hu J., Li L., Zhang D., Liang W. (2023). THERMOSENSITIVE BARREN PANICLE (TAP) is required for rice panicle and spikelet development at high ambient temperature. New Phytol..

[53] Chen C., Begcy K., Liu K., Folsom J.J., Wang Z., Zhang C., Walia H. (2016). Heat stress yields a unique MADS box transcription factor in determining seed size and thermal sensitivity. Plant Physiol..

[54] Liu W., Yin T., Zhao Y., Wang X., Wang K., Shen Y., Ding Y., Tang S. (2021). Effects of High Temperature on Rice Grain Development and Quality Formation Based on Proteomics Comparative Analysis Under Field Warming. Front. Plant Sci..

[55] Yan H., Wang C., Liu K., Tian X. (2021). Detrimental effects of heat stress on grain weight and quality in rice (*Oryza sativa L*.) are aggravated by decreased relative humidity. PeerJ.

[56] Gann P.J., Esguerra M., Counce P.A., Srivastava V. (2021). Genotype-dependent and heat-induced grain chalkiness in rice correlates with the expression patterns of starch biosynthesis genes. Plant Environ. Interact..

[57] Li B., Cai H., Liu K., An B., Wang R., Yang F., Zeng C., Jiao C., Xu Y. (2023). DNA Methylation Alterations and Their Association with High Temperature Tolerance in Rice Anthesis. J. Plant Growth Regul..

[58] Chakraborty A., Wylie S.J. (2025). CRISPR/Cas9 for Heat Stress Tolerance in Rice: A Review. Plant Mol. Biol. Report..

[59] Zhang Y., Zou B., Lu S., Ding Y., Liu H., Hua J. (2016). Expression and promoter analysis of the OsHSP16.9C gene in rice. Biochem. Biophys. Res. Commun..

[60] Xuan Q., Wang J., Nie Y., Fang C., Liang W. (2024). Research Progress and Application of Miniature CRISPR-Cas12 System in Gene Editing. Int. J. Mol. Sci..

[61] Lou H., Li S., Shi Z., Zou Y., Zhang Y., Huang X., Yang D., Yang Y., Li Z., Xu C. (2025). Engineering source-sink relations by prime editing confers heat-stress resilience in tomato and rice. Cell.

[62] Li X., Xie J., Dong C., Zheng Z., Shen R., Cao X., Chen X., Wang M., Zhu J.K., Tian Y. (2024). Efficient and heritable A-to-K base editing in rice and tomato. Hortic. Res..

[63] Hu C., Jiang J., Li Y., Song S., Zou Y., Jing C., Zhang Y., Wang D., He Q., Dang X. (2022). QTL mapping and identification of candidate genes using a genome-wide association study for heat tolerance at anthesis in rice (*Oryza sativa* L.). Front. Genet..

[64] Liu X., Ji P., Liao J., Duan X., Luo Z., Yu X., Jiang C.J., Xu C., Yang H., Peng B. (2025). CRISPR/Cas knockout of the NADPH oxidase gene OsRbohB reduces ROS overaccumulation and enhances heat stress tolerance in rice. Plant Biotechnol. J..

[65] Li P., Jiang J., Zhang G., Miao S., Lu J., Qian Y., Zhao X., Wang W., Qiu X., Zhang F. (2022). Integrating GWAS and transcriptomics to identify candidate genes conferring heat tolerance in rice. Front. Plant Sci..

[66] Pan Y.H., Chen L., Zhu X.Y., Li J.C., Rashid M.A.R., Chen C., Qing D.J., Zhou W.Y., Yang X.H., Gao L.J. (2023). Utilization of natural alleles for heat adaptability QTLs at the flowering stage in rice. BMC Plant Biol..

[67] Grenier C., Cao T.V., Ospina Y., Quintero C., Chatel M.H., Tohme J., Courtois B., Ahmadi N. (2015). Accuracy of Genomic Selection in a Rice Synthetic Population Developed for Recurrent Selection Breeding. PLoS ONE.

[68] Zhang Y., Zhang M., Ye J., Xu Q., Feng Y., Xu S., Hu D., Wei X., Hu P., Yang Y. (2023). Integrating genome-wide association study into genomic selection for the prediction of agronomic traits in rice (*Oryza sativa* L.). Mol. Breed..

[69] Feng B., Xu Y., Fu W., Li H., Li G., Li J., Wang W., Tao L., Chen T., Fu G. (2023). RGA1 Negatively Regulates Thermo-tolerance by Affecting Carbohydrate Metabolism and the Energy Supply in Rice. Rice.

[70] El-Esawi M.A., Alayafi A.A. (2019). Overexpression of Rice Rab7 Gene Improves Drought and Heat Tolerance and Increases Grain Yield in Rice (*Oryza sativa* L.). Genes.

[71] Guo Z., Zuo Y., Wang S., Zhang X., Wang Z., Liu Y., Shen Y. (2024). Early signaling enhance heat tolerance in Arabidopsis through modulating jasmonic acid synthesis mediated by HSFA2. Int. J. Biol. Macromol..

[72] Huang J., Gao L., Luo S., Liu K., Qing D., Pan Y., Dai G., Deng G., Zhu C. (2022). The genetic editing of GS3 via CRISPR/Cas9 accelerates the breeding of three-line hybrid rice with superior yield and grain quality. Mol. Breed..

[73] Li N., Zhao Y., Han J., Yang Q., Liang J., Liu X., Wang Y., Huang Z. (2024). Impacts of future climate change on rice yield based on crop model simulation-A meta-analysis. Sci. Total Environ..

[74] Yang Y., Yu J., Qian Q., Shang L. (2022). Enhancement of Heat and Drought Stress Tolerance in Rice by Genetic Manipulation: A Systematic Review. Rice.

[75] Tong J., Ren Z., Sun L., Zhou S., Yuan W., Hui Y., Ci D., Wang W., Fan L.M., Wu Z. (2022). ALBA proteins confer thermotolerance through stabilizing HSF messenger RNAs in cytoplasmic granules. Nat. Plants.

